# Omental adipose tissue fibrosis and insulin resistance in severe obesity

**DOI:** 10.1038/nutd.2015.22

**Published:** 2015-08-10

**Authors:** V Guglielmi, M Cardellini, F Cinti, F Corgosinho, I Cardolini, M D'Adamo, M C Zingaretti, A Bellia, D Lauro, P Gentileschi, M Federici, S Cinti, P Sbraccia

**Affiliations:** 1 Department of Systems Medicine, Laboratory of Molecular Medicine, University of Rome ‘Tor Vergata', Rome, Italy; 2 Obesity Center (EASO accredited COM), Policlinico Tor Vergata, Rome, Italy; 3 Department of Experimental and Clinical Medicine, Obesity Center, University of Ancona (Politecnica delle Marche), Ancona, Italy; 4 CAPES Foundation, Ministry of Education of Brazil, Brasília, DF, Brazil; 5 Department of Experimental Medicine and Surgery, University of Rome ‘Tor Vergata', Rome, Italy

## Abstract

**Background/Objectives:**

The unresolved chronic inflammation of white adipose tissue (WAT) in obesity leads to interstitial deposition of fibrogenic proteins as reparative process. The contribution of omental adipose tissue (oWAT) fibrosis to obesity-related complications remains controversial. The aim of our study was to investigate whether oWAT fibrosis may be related to insulin resistance in severely obese population.

**Subjects/Methods:**

Forty obese subjects were studied by glucose clamp before undergoing bariatric surgery and thus stratified according to insulin resistance severity (*M*-value). From the first (Group B: *n*=13; M=1.9±0.7 mg kg^−1^ min^−1^) and the highest (Group A: *n*=14; M=4.5±1.4 mg kg^−1^ min^−1^) *M*-value tertiles, which were age-, waist- and body mass index-matched, oWAT samples were then obtained.

Gene expression of collagen type I, III and VI, interleukin-6, profibrotic mediators (transforming growth factor (TGF)-β1, activin A, connective tissue growth factor), hypoxia inducible factor-1α (HIF-1α) and macrophage (CD68, monocyte chemotactic protein (MCP)-1, CD86, CD206, CD150) markers were analyzed by quantitative reverse transcription PCR. Adipocyte size and total fibrosis were assessed by histomorphometry techniques.

**Results:**

Fibrosis at morphological level resulted significantly greater in Group B compared with Group A, although collagens gene expression did not differ. Notably, collagen VI messenger RNA significantly correlated with collagen I, collagen III, HIF-1α, TGF-β1, CD68, MCP-1 and CD86 transcription levels, supporting their relation with fibrosis development.

**Conclusions:**

In conclusion, we show for the first time that human oWAT fibrosis in severe obesity is consistent with a higher degree of insulin resistance measured by glucose clamp. Therefore, collagen deposition could represent a maladaptive mechanism contributing to obesity-related metabolic complications.

## Introduction

The role of white adipose tissue (WAT) in the pathogenesis of insulin resistance and metabolic syndrome has been extensively studied.

Particularly, omental adipose tissue (oWAT) represents the determinant marker of obesity-related metabolic risk and correlates with the development of insulin resistance.^1^ One pathophysiological explanation is oWAT unique anatomic location. Indeed, this fat depot directly influences lipid and glucose metabolism through the release of free fatty acids and adipokines into the portal system up to the liver.^2^

To accommodate the changes induced in WAT by prolonged positive energy balance, WAT extracellular matrix (ECM) remodeling, which has a pivotal role in adipogenesis^3^ and tissue architecture,^4^ occurs by degradation of existing ECM and synthesis of new ECM components, as part of a regenerative process, in response to inflammation. In the absence of resolution of inflammation, fibrogenic cellular actors remain activated and fibrillar components persist and accumulate.^5, 6^

Collagens are the most abundant proteins of interstitial fibers and pericellular basement membranes in WAT. Among these components, collagen type I and III are more frequently observed in fibrous bundles, providing the major ECM framework, whereas collagen type VI surrounds parenchymal adipocytes having a pivotal role in ECM stability.^7^

The WAT-secreted ECM proteins seem to have an important role in the complex dynamic of weight gain. Higher amounts of total and pericellular fibrosis were found, respectively, in oWAT and in both oWAT and subcutaneous adipose tissue (scWAT) of obese compared with lean subjects.^8^ In human scWAT, messenger RNA (mRNA) of collagen type VI α3-subunit (col6a3) positively correlated with body mass index, total body fat mass and, in obese, with oWAT amount.^9^ Moreover, interestingly, col6a3 mRNA expression was lower in scWAT than in oWAT.^9^

Mice lacking col6a3 (col6KO), characterized by impaired WAT fibrosis and ECM stability, on challenging (high-fat diet or *ob/ob* background) showed reduced WAT inflammation, adipocytes endoplasmic reticulum stress and death, and improved lipid and glucose metabolism compared with wild-type mice, despite severe obesity and larger adipocytes in scWAT.^10^ Accordingly, a mirroring model of excessive ECM accumulation, due to genetic ablation of collagenase membrane type 1-matrix metalloproteinase, which plays a major role in collagen I degradation, demonstrated increased fibrosis around smaller adipocytes and developed severe metabolic complications,^11^ suggesting that ECM deposition might contribute to restrain adipose tissue expandability. The role of collagen fibers and adhesion proteins (fibronectin) in regulating adipocytes morphology and function has been addressed also in a three-dimensional culture system where bio-mechanical cues, partly inducing adipocyte deformation, affected lipolytic activity and secretory functions.^12^

In humans, the metabolic consequences of WAT fibrosis remain controversial. Indeed, abnormal collagen deposition, a hallmark of fibrosis development in WAT, was found increased in scWAT of Asian Indians, models of high susceptibility to insulin resistance, than in BMI-matched Caucasians^10^ and tightly associated with tissue infiltration of macrophages and other immune cells;^13^ contrariwise, oWAT fibrosis was positively correlated with a protective serum lipid pattern.^8^

Furthermore, previous human studies have not dissected the impact of fibrosis accumulation on insulin sensitivity (S_I_) from that of increasing BMI.^14^

Thus, although during the development of obesity the ECM synthesis is a well-established physiological response, whether in human obesity it correlates with a higher degree of insulin resistance independent of either BMI or central adiposity, still needs to be clearly defined.

To address this issue, we studied the expression of several collagen types and total fibrosis at histological level in oWAT in severely obese subjects, with relation to insulin resistance severity assessed by glucose clamp. Moreover, we investigated the correlations between oWAT fibrosis and the transcriptional levels of profibrotic factors, hypoxia and macrophage M1 and M2 phenotype markers.

## Subjects and methods

### Study population and procedures

Between 2011 and 2012 we studied 40 severely obese subjects (sex 28 F/12 M; age 40.3±9.2 year; BMI 48.8±7.4 Kg m^−^^2^) attending the outpatient service of the Obesity Center of the University Hospital ‘Policlinico Tor Vergata' (Rome, Italy), with no clinical history of type 2 diabetes (T2D), undergoing bariatric surgery (Sleeve Gastrectomy). Thus, all recruited patients met the criteria for bariatric surgery, whereas the exclusion criteria were: acute or chronic inflammatory or infectious disease, malignant neoplasms and alcohol intake >20 g daily. Before surgery, the patients underwent a comprehensive medical evaluation including clinical history, anthropometric examination (BMI, waist and hip circumference) and blood pressure assessment, blood samples were withdrawn for biochemical analysis and an oral glucose tolerance test (OGTT) and hyperinsulinemic euglycemic clamp were performed.

Subjects were then stratified according to insulin resistance (glucose clamp), and from the highest (Group A: *n*=14; M=4.5±1.4 mg kg^–1^ min^−1^) and lowest (Group B: *n*=13; M=1.9±0.7 mg kg^−1^ min^−1^) *M*-value tertiles oWAT samples were then obtained during bariatric surgery. Although, as mentioned above, no patient at the time of the enrollment had a past clinical history of T2D or was treated with antidiabetic drugs, the OGTT revealed that in Group A, two patients had impaired glucose tolerance (IGT) and one was overtly diabetic, whereas in Group B, four patients were IGT and three diabetics.

Weight and height were measured after the overnight fasting, and BMI was calculated as weight (kg) divided by height (m) squared. Readings of clinic blood pressure were obtained in the left arm of the supine patients, after 5 min of quiet rest, with a mercury sphygmomanometer.

For all these parameters, mean values were determined from two independent measurements.

The study protocol was approved by the ethical committee of ‘Policlinico Tor Vergata' and the investigation was conducted in accordance with the Declaration of Helsinki. All patients gave a written informed consent after individual explanation.

### Laboratory assays

After a 12-h overnight fast, the patients underwent biochemical tests and an OGTT. Fasting glucose, total-cholesterol, high-density lipoprotein and low-density lipoprotein cholesterol, triglycerides, creatinine, urea, aminotransferases and gamma-glutamyltransferase were assessed by standard immunoenzymatic methods; insulin levels were measured by immunoradiometric assay, whereas Hemoglobin A1C percentage by high-performance liquid chromatography method; high-sensitivity C-reactive protein was measured by latex agglutination. A standard 75-g OGTT was performed: venous blood samples were obtained in the fasting state and 30, 60, 90 and 120 min after glucose ingestion for the assessment of blood glucose and insulin concentrations. The total area under the curve of glucose and insulin responses during the 2-h OGTT was calculated using a trapezoidal model. Normal glucose tolerance (NGT), IGT and impaired fasting glucose were diagnosed according to American Diabetes Association criteria.^15^

On a second occasion (at least 1 week after the initial evaluation), after a 12-h overnight fast, subjects underwent an euglycemic–hyperinsulinemic clamp to evaluate insulin sensitivity. Insulin (Humulin-R; Eli Lilly, Indianapolis, IN, USA) was given as a primed continuous infusion targeted to produce plasma insulin levels of ∼80 μU ml^−1^. Thereafter, the insulin infusion rate was fixed at 40 mU m^−^^2^ min^−1^. The blood glucose level was constantly maintained at ~90 mg dl^−1^ for the next 120 min, by infusing 20% glucose at varying rates according to blood glucose measurements performed at 5-min intervals (the mean coefficient of variation of blood glucose was <4%). The glucose disposal during the clamp (*M*-value) was expressed as the amount of glucose infused per kg of body weight per minute during the last 60 min of the study.

### Adipose tissue collection

oWAT samples (average 0.5–1.0 g) obtained during bariatric surgery were divided into two portions. The first portion was immediately embedded in RNA later (Applied Biosystems, Foster City, CA, USA) and then placed in empty RNase-DNase-free Eppendorf tubes, snap-frozen in liquid nitrogen and stored at −80 °C for RNA isolation. The second fragment of each collected tissue sample was fixed in 4% formalin overnight at 4 °C and then paraffin embedded for morphological studies.

### Gene expression analysis by quantitative real-time PCR

Total RNA from human WAT samples was extracted using RNeasy Lipid Tissue Mini kits (Qiagen). Two micrograms of total RNA were reverse-transcribed into complementary DNA using the High Capacity cDNA Archive kit (Applied Biosystems). Fifty nanograms of complementary DNA were amplified by real-time PCR using an ABI PRISM 7500 System and TaqMan reagents (Applied Biosystems) and normalized to 18 S ribosomal RNA as an endogenous control. Each reaction was performed in triplicate, and the relative gene copy number was calculated as 2^−ΔΔCt^. Transcripts encoding for collagen type I (col1a1), III (col3a1) and VI (col6a3), profibrotic mediators (transforming growth factor (TGF)-1β activin A; connective tissue growth factor), hypoxia (hypoxia inducible factor (HIF)-1α), inflammation (interleukin-6), macrophage infiltration (CD68; monocyte chemotactic protein (MCP)-1) and polarization (M1: CD86; M2: CD206, CD150) markers were analyzed. PCR primers and TaqMan probes were obtained from Applied Biosystems and optimized according to manifacturer's protocol.

### Histomorphometry

Adipocyte size was determined in hematoxylin/eosin-stained sections as the mean cell area (in μm^2^) of 300 random adipocytes/subject on digital images acquired at × 20 by a Nikon Eclipse e800 light microscope (Nikon, Tokyo, Japan) using a digital image system (LUCIA Imaging, v 4.82, Czech Republic).

Fibrosis was localized by picrosirius red staining^5^ in × 40 digital images and quantified profiling the picrosirius red-stained areas in multiple fields using the measure tool of the LUCIA image analysis. The total fibrosis was expressed as the ratio of fibrous tissue area stained with picrosirius red/total tissue surface, as described.^6^

### Statistical analysis

Statistical analysis was performed with SPSS 19.0 software (SPSS, Chicago, IL, USA). Descriptive statistics were given by means±s.d. The Kolmogorov–Smirnov test was used to verify quantitative variables for normality distribution. Comparisons between two groups (Group A versus Group B and NGT versus IGT+T2D) were determined by using non parametric Mann–Whitney test for unpaired data. Kruskal–Wallis test followed by Dunn's *post hoc* test was used to compare the low, medium and high adipocyte area tertiles. Relationships between variables were evaluated using the non parametric Spearman's correlation test for non normal variables. For all these analysis a *P*-value <0.05 based on two-sided test was considered statistically significant.

## Results

### Patient's characteristics

Forty severely obese subjects underwent OGTT and glucose clamp before bariatric surgery. We stratified the subjects according to tertiles of insulin resistance (estimated by glucose clamp) and studied the highest (Group A: *n*=14) and the lowest (Group B: *n*=13) *M*-value tertiles, so that Group A and B, respectively, represented the less and more insulin-resistant groups. Group A and B were age-, waist circumference- and BMI-matched. Clinical and biochemical parameters of Group A and B are presented in Table 1.

Subjects with NGT (normal glycemia according to both fasting and 2- h glucose) (*n*=17) differed from IGT and/or T2D (IGT+T2D: *n*=10) only in glucose area under the curve (NGT: 867±160 mgmindl^−1^, IGT+T2D: 1082±133.4 mgmindl^−1^; *P*<0.01) and fasting plasma insulin (NGT: 29±18.9 μU ml^−1^, IGT+T2D: 46.5±25.6 μU ml^−1^; *P*<0.05) (Supplementary Table).

### oWAT fibrosis is consistent with a higher degree of insulin resistance

**Figure 1:**
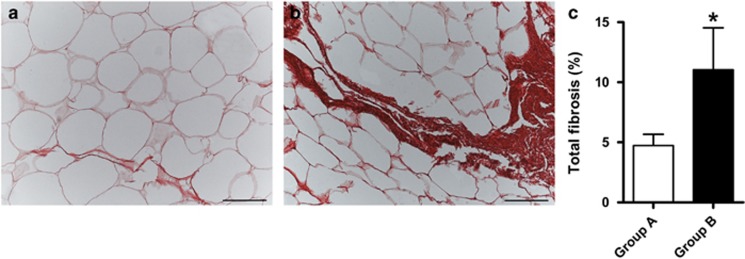
Total fibrosis in oWAT sections. Light microscopy. Picrosirius Red staining. Representative images of oWAT of Group A (**a**) and Group B (**b**). Bar: 100 μm. (**c**) Total fibrosis amount (expressed as fibrous tissue area stained with picrosirius red/total tissue surface ratio) in Group A (*n*=12) and B (*n*=11). Data are presented as mean ± s.e.m. Mann–Whitney test * *P*<0.05.

**Figure 2:**
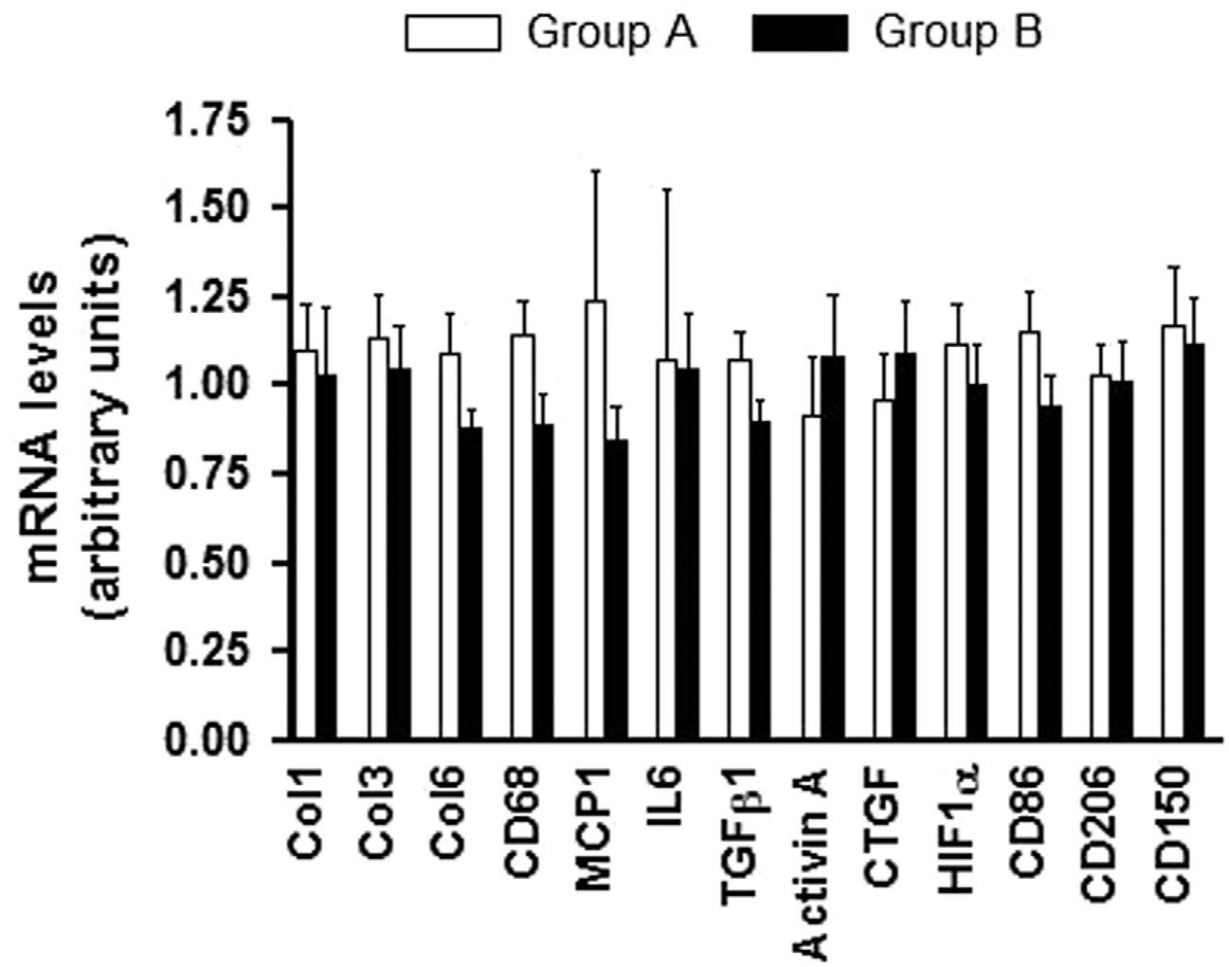
Gene expression levels in Group A and Group B.

Fibrosis quantification at tissue level was significantly higher in Group B compared with Group A (A: 4.7±2.9% B: 11±11.5% *P*<0.05) (Figure 1), although gene expression of collagens did not differ, as well as the expression of fibrogenic mediators, inflammatory and macrophage markers (Figure 2).

Nevertheless, only in Group B collagens expression levels were associated with serum concentrations of total-cholesterol (Col I: *r*=0.72, *P*<0.05; Col III: *r*=0.59, *P*<0.05), low-density lipoprotein cholesterol (Col I: *r*=0.86, *P*<0.0001; Col III: *r*=0.75, *P*<0.01; Col VI: *r*=0.67, *P*<0.01) and triglycerides (Col I: *r*=0.66, *P*<0.05).

We found no difference in fibrosis amount or collagens expression between obese NGT and IGT+T2D patients.

### Collagens in obese oWAT correlate with profibrotic factors, tissue hypoxia and macrophages infiltration

**Figure 3:**
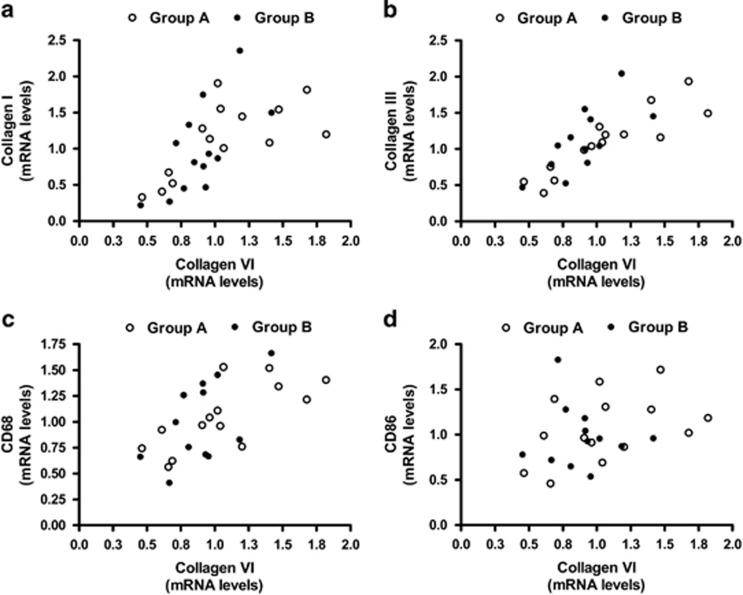
Correlations between transcription levels of collagens and macrophage markers. (**a**) Collagen VI mRNA correlates with collagen type I (*r*=0.72, *P*<0.0001), (**b**) collagen type III (*r*=0.82, *P*<0.0001), (**c**) CD68 (*r*=0.59, *P*<0.001) and (**d**) CD86 (*r*=0.38, *P*=0.04) transcription levels.

Irrespective of insulin resistance severity, mRNA levels of collagens correlated reciprocally (Figure 3a and b) and with macrophage markers CD68 (Col I: *r*=0.4, *P*<0.01; Col III: *r*=0.47, *P*<0.01; Col VI: *r*=0.59, *P*<0.05) (Figure 3c) and MCP-1 (Col III: *r*=0.38, *P*<0.05; Col VI: *r*=0.4, *P*<0.05). To characterize the oWAT macrophages, mRNA levels of CD86 (M1-phenotype marker), CD206 and CD150 (M2-phenotype markers) were analyzed. Collagens were positively correlated with CD86 (Col III: *r*=0.43, *P*<0.05; Col VI: *r*=0.38, *P*<0.05) but not with CD206 and CD150 (Figure 3d).

**Figure 4:**
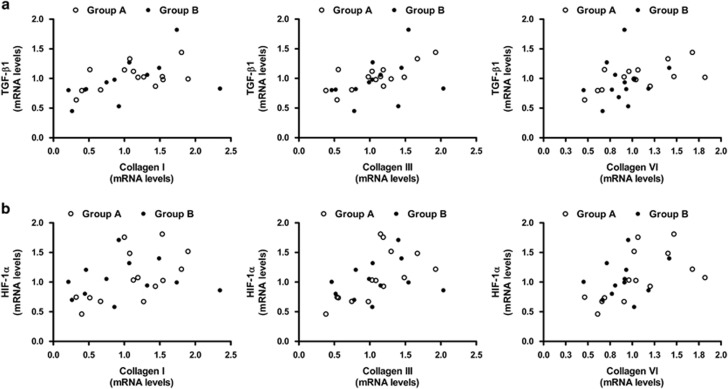
Correlations between transcription levels of collagens and profibrotic factors. (**a**) Collagens (I, III and VI) expression correlates with TGF-β1 (Col I: *r*=0.58, *P*=0.02; Col III: *r*=0.54, *P*=0.004; Col VI: *r*=0.48, *P*=0.01) and (**b**) HIF-1α (Col I: *r*=0.42, *P*=0.03; Col III: *r*=0.6, *P*=0.002; Col VI: *r*=0.62, *P*=0.001) mRNA levels.

Of note, collagens (Col I, III and VI) correlated with the expression of TGF-β1 (Col I: *r*=0.58, *P*<0.01; Col III: *r*=0.54, *P*<0.01; Col VI: *r*=0.48, *P*<0.05) (Figure 4a) and the hypoxia marker HIF-1α (Col I: *r*=0.42, *P*<0.05; Col III: *r*=0.6, *P*<0.01; Col VI: *r*=0.62, *P*<0.01) (Figure 4b). Collagen I correlated also with activin A mRNA levels (*r*=0.4, *P*<0.05).

Besides, TGF-β1 was significantly correlated with HIF-1α (*r*=0.41, *P*<0.05) and connective tissue growth factor (*r*=0.55, *P*<0.01) highlighting the network among profibrotic factors. It correlated also with markers of M1 macrophage infiltration (CD68: *r*=0.58, *P*<0.01; CD86: *r*=0.45, *P*<0.05; CD206: *r*=0.6, *P*<0.01).

Group A and B did not differ in mean adipocyte area (A: 3384±693 μm^2^; B: 3722±902 μm^2^, *P*=NS). Accordingly, adipocyte size was not significantly correlated with collagen (Col I, III and VI) mRNA levels, fibrosis amount and profibrotic factors.

As expected, the high tertile of adipocyte area was characterized by significantly greater CD68 (low: 0.82±0.07, high: 1.17±0.1; *P*<0.05) and CD206 (low: 0.84±0.08, high: 1.25±0.1; *P*<0.05) expression levels compared with the low tertile.

## Discussion

This is the first study designed to explore oWAT collagens expression and fibrosis at tissue level in severely obese subjects in relation to significantly different degrees of insulin resistance assessed by glucose clamp. Indeed, we performed a detailed analysis of col1a1, col3a1 and col6a3 expression and quantification of total fibrosis at morphological level in oWAT of age-, BMI- and waist-matched obese patients, grouped according to insulin resistance severity (estimated by glucose clamp), or alternatively glucose tolerance.

Although a causal impact of WAT fibrosis accumulation on obesity-related complications is difficult to evaluate because of the correlative nature of the investigation in human studies, our data suggest that oWAT fibrosis is consistent with a higher degree of insulin resistance in human obesity and confirm *in vivo* the link of oWAT collagens with profibrotic mediators, hypoxia and macrophage markers. In contrast, transcriptional levels of collagens did not differ in more and less insulin-resistant subjects, although only in the latter were suggestively associated with the serum lipids.

Unlike mouse WAT in which collagen type VI is most abundantly expressed,^10^ no difference in the transcription levels of collagens type I, III and VI in human oWAT was detected.

Discrepancies between collagens expression and morphology have been already described.^16^ We may hypothesize that the ECM increase depends on collagen enhanced stabilization and cross-linking^12, 17^ that can prevent the ECM physiological degradation.

What turns oWAT fibrotic response from representing an adaptive physiological process to being detrimental is so far mainly matter of speculation. Intriguingly, in oWAT samples we observed adipocytes entrapped in the fibrotic bundles, not surrounded by macrophages. Some were perilipin-negative adipocytes evocative of dysfunctional or dying adipocytes described in crown-like structure.^8, 18^ On the basis of these observations, we may speculate that oWAT fibrosis negatively impacts on insulin sensitivity by making the adipocytes dysfunctional.^19^ Consistent with col6KO mouse model, collagen deposition contributes to WAT dysfunction by impairing WAT expansion, lipid storage flexibility and by promoting ectopic accumulation of lipids over local storage.^9, 10^ Interactions between ECM and adipocyte membranes could trigger the β1-integrin/extracellular signal-regulated kinase signaling pathway and thus pyroptosis,^5, 20^ and directly affect insulin sensitivity by modifying the arrangement of membrane caveolae where insulin receptors are localized.^10^ The mechanical deformation of adipocytes in a three-dimensional culture system decreased lipolysis and enhanced the expression of fibro-inflammatory mediators and endoplasmic reticulum stress markers.^12^

The reported association between oWAT fibrosis and obesity-related insulin resistance appears independent of diabetes in line with a previous report.^9^ In fact, fibrosis amount and collagen expression in NGT subjects were similar to those with IGT and/or T2D (BMI and waist-matched). This observation suggests that oWAT fibrosis does not directly contribute to the development of T2D.

The obese groups (NGT and IGT+T2D) were similarly insulin resistant but only the diabetics had β-cell failure, which might not relate with oWAT fibrosis.

In our study sample, oWAT fibrosis and adiposity were not significantly related, conceivably because both BMI and body fat partitioning (waist and hip circumference) were quite homogeneous.

Collagens expression was positively correlated with CD68 and MCP-1, which are known to be negatively related to insulin sensitivity.^21^

In our study population, collagens were uniquely correlated with the expression of CD86 (M1 macrophage marker), which usually identifies the crown-like structure macrophages,^22^ but not with CD206 and CD150 (M2 macrophage markers), despite the latters were described as co-locating with collagen VI in fibrotic areas.^14^ After all, M2 macrophages not only modulate tissue remodeling and repair by secreting profibrotic factors^23, 24^ but could also limit collagen deposition as found in overweight children.^16^ The correlations between col6a3 and local inflammation based on MCP-1 expression in human scWAT^9^ and the impaired macrophage infiltration in col6KO mice^10^ support our findings.

This underlines the interconnection between inflammation and fibrosis within WAT, although their causal and temporal relationship in the development of obesity still needs to be defined.^7^ Indeed, overfeeding increased both inflammation and col6a3 expression in WAT^9, 17^ and proinflammatory factors enabled preadipocytes to synthetize ECM components.^25^

Differently, systemic inflammation based on C-reactive protein levels were not related to oWAT fibrosis.

The overall mean adipocyte area was not correlated either with oWAT collagens expression or with fibrosis amount, as previously described in human scWAT.^9^ However, we cannot exclude a relationship between fibrosis and smaller adipocyte size in a more comprehensive analysis of oWAT, in regard of the notion that WAT fibrosis is not a homogeneous phenomenon.^14^

All collagens were correlated with TGF-β1 and HIF-1α expression. TGF-β1 which is enhanced in human obesity and primarily released by WAT macrophages,^26^ which also secrete matrix metalloproteinase-9 responsible for the proteolytic activation of its latent form,^27^ is implicated in ECM homeostasis^28^ and fibrosis.^29, 30, 31^ Also, the upregulation of HIF-1α observed in hypoxic conditions could represent a driving phenomenon for WAT fibrosis development. We also confirmed that activin A, secreted by fibro-inflammatory human preadipocytes in culture,^25^ is positively associated *in vivo* with collagen expression.

The relationship among profibrotic factors (for example, TGF-β1 and connective tissue growth factor) suggests a complex fibrogenic cascade. Indeed, connective tissue growth factor is a gene target of both TGF-β1 and HIF-1α.^17, 32^ It is regulated by weight changes,^12^ increases along with collagen III expression and has a role in WAT fibro-inflammation via mechano-transduction pathways.^12^

Finally, we acknowledge some study limitations, mainly related to the sole transcriptional analysis of profibrotic mediators and macrophage markers, which could not reflect the relative protein levels and does not take into account the primarily post-translational regulation of HIF-1α under hypoxic conditions.^33^

In conclusion, our data show that oWAT fibrosis is significantly increased in obese subjects with higher degree of insulin resistance assessed by glucose clamp, whereas it was not affected by glucose intolerance or diabetic status. Moreover, our study confirms *in vivo* the link between oWAT fibrosis, profibrotic mediators, hypoxia and macrophage infiltration markers.

## Figures and Tables

**Table 1:** Characteristics of Group A and Group B

	*Group A*	*Group B*	P*-value*
*N*	14	13	
Age (year)	40.5±9.3	43.3±8.1	
Sex (M/F)	3 /11	5/8	
BMI (Kg m^−^^2^)	46.8±6.8	50.2±7.6	
Smokers (*n*)	8	8	
*M*-value (mg kg^−1^ min^−1^)	4.5±1.4	1.9±0.7	<0.001
Waist circumference (cm)	126.8±22	135±14	
Hip circumference (cm)	130.7±30	141.5±16	
Systolic BP (mmHg)	141.7±32.2	140.3±16.6	
Diastolic BP (mmHg)	88.9±15.3	90.3±12.4	
IFG/IGT/T2D (*n*)	0/2/1	0/4/3	
Fasting glucose (mg dl^−1^)	93.5±10.1	102±22.3	
HbA1c % (mmol mol^−1^)	5.7±0.3 (39± 3.3)	6±0.4 (42±4.4)	
Glucose AUC (mg min dl^−1^)	890.3±161.5	1001±192.2	
Insulin AUC (μU min ml^−1^)	11936±6223	25022±12257	<0.01
Total cholesterol (mg ml^−1^)	202.8±32	187.3±34.7	
HDL cholesterol (mg ml^−1^)	47.3±12.4	42.7±10.6	
Triglycerides (mg dl^−1^)	113.4±39.1	142±41.8	<0.05
AST (mg dl^−1^)	17.8±8.4	41.9±32	<0.01
ALT (mg dl^−1^)	38.3±12.9	57.3±27.2	<0.05
γGT (mg dl^−1^)	33.6±13	51.1±22.8	<0.05
hsCRP (mg l^−1^)	0.9±0.5	1.5±2.1	
ASA/statins (*n*)	0	0	
β-b/ACE-I/ARBs (*n*)	0/0/1	0/1/2	

Abbreviations: ACE-I, angiotensin-converting enzyme inhibitors; ALT, alanine aminotransferase; ARBs, angiotensin receptors blockers; AST, aspartate aminotransferase; ASA, acetyl-salicylic acid; AUC, area under the curve; β-b, beta-blockers; BP, blood pressure; BMI, body mass index, hsCRP, high-sensitivity c-reactive protein; γGT, gamma-glutamyltransferase; IFG, impaired fasting glucose; IGT, impaired glucose tolerance; HbA1c, haemoglobin A1C; T2D, type 2 diabetes. Clinical and biochemical features of Group A and B. Data are presented as mean±s.d.
