# Supplementary material for: Omental adipose tissue fibrosis and insulin resistance in severe obesity
Source: Nutr Diabetes. 2015 Aug 10;5(8):e175–. doi: 10.1038/nutd.2015.22 (PMC4558556; doi:10.1038/nutd.2015.22)
Supplement: Supplementary Table [file nutd201522x1.doc]

**S1 Table. Characteristics of** NGT and IGT+T2D subjects.

|  | **NGT** | **IGT+T2D** | ***p value*** |
| --- | --- | --- | --- |
| N | 17 | 10 |  |
| Age (y) | 40.7±8.7 | 43.7±8.9 |  |
| Sex (M/F) | 4/13 | 4/6 |  |
| BMI (Kg/m^2^) | 48.6±6.8 | 48.2±8.4 |  |
| M (mg·kg^-1^·min^-1^) | 3.9±1.8 | 2.3±1 |  |
| Waist circumference (cm) | 132.7±21.5 | 133.8±14 |  |
| Hip circumference (cm) | 136.6±30.6 | 135.4±9.5 |  |
| Systolic BP (mmHg) | 138.8±30.3 | 145±14.3 |  |
| Diastolic BP (mmHg) | 88.5±15.7 | 91.5±10 |  |
| Fasting glucose (mg/dl) | 92.3±17.8 | 105.6±22.5 |  |
| Fasting Insulin (µU/ml) | 28.9±19 | 43.5±25.6 | *p<0.05* |
| HbA1c (%) | 5.7±0.3 | 6.2±0.4 |  |
| Glucose AUC (mg·min/dl) | 867±160 | 1082±133.4 | *p<0.01* |
| Insulin AUC (µU·min/ml) | 16525±8529 | 20714±15793 |  |
| Total Cholesterol (mg/ml) | 202.2±31.6 | 183.7±36.2 |  |
| HDL Cholesterol (mg/ml) | 49.1±11.7 | 38.3±12.7 |  |
| Triglycerides (mg/dl) | 106±25.4 | 162.5±38.2 |  |
| AST (mg/dl) | 24.5±30 | 38.9±19.2 |  |
| ALT (mg/dl) | 44.2±26 | 53±216.8 |  |
| γGT (mg/dl) | 38.6±21 | 47.9±16.7 |  |
| hsCRP (mg/l) | 1.4±1.8 | 0.7±0.7 |  |

Clinical and biochemical features of NGT and IGT+T2D subjects.

*AUC*, area under the curve; *BP*, blood pressure; *IFG*, impaired fasting glucose; *IGT*, impaired glucose tolerance; *NGT*, normal glucose tolerance; *T2D*, type 2 diabetes. IGT+T2D group includes subjects with IGT and/or T2D. Data are presented as mean±SD.
